# Designing a Robust and Versatile System to Investigate Nutrient Exchange in, and Partitioning by, Mycorrhiza (*Populus x canesces* x *Paxillus involutus*) Under Axenic or Greenhouse Conditions

**DOI:** 10.3389/ffunb.2022.907563

**Published:** 2022-06-17

**Authors:** Katharina Schreider, Jens Boy, Leopold Sauheitl, Aline Fernandes Figueiredo, Alberto Andrino, Georg Guggenberger

**Affiliations:** ^1^ Institute of Soil Science, Leibniz Universität Hannover, Hannover, Germany; ^2^ Institute of Physiology and Cell Biology, Tierärztliche Hochschule Hannover, Hannover, Germany

**Keywords:** ectomycorrhizal, *in vitro*, laboratory protocols, mesocosm, *Paxillus involutus*, *Populus x canscens*, rhizotrone, phosphorus availability

## Abstract

Phosphorus (P) bioavailability affects plant nutrition. P can be present in soils in different chemical forms that are not available for direct plant uptake and have to be acquired by different mechanisms, representing different resource niches. These mechanisms, of which many seem to be attributed to mycorrhiza, likely influence the diversity and stability of plant communities in natural ecosystems, as they also might help to overcome a future shortage of P supply in agro-ecosystems. In order to understand the mechanisms of P acquisition, the associated carbon costs, and the resource partitioning by mycorrhizal fungi, the ecosystem situation has to be mimicked in smaller scaled experiments. Here, different experimental setups are evaluated using plantlets of *Populus x canescens* and its functional ectomycorrhizal (ECM) fungus *Paxillus involututs* strain MAJ. To investigate resource partitioning involving mycorrhizae, the protocols of this study describe preparation of an *in vitro* and a rhizotrone culture systems for studies under axenic conditions as well as a mesocosm culture system for greenhouse conditions. We also describe the construction of separate compartments containing nutrients and excluding plant roots as well as the progress that has been made in *in vitro* propagation of plant and ECM fungal material. The practical experience made in our study shows that the *in vitro* culture system is prone to desiccation and its construction and maintenance are more time consuming and complicated. In contrast, with the axenic rhizotrone culture system and the mesocosms we have created more robust and very versatile systems that are also suitable for greenhouse conditions.

## Highlight

Compartmental culture systems have been developed to study the mycorrhizal associations to investigate the mycorrhizal fungal mediated P resource partitioning and plant P uptake, required host carbon investment and mycorrhizal fungal acquisition mechanisms in dependence of P form bioavailability.

## Introduction

More than 90% of phosphorus (P) in the soil is present in different chemical forms that are unavailable to plants (Mengel et al., 2001). The association of plants with mycorrhizal fungi can increase the bioavailability of P (Hinsinger, 2001; Hinsinger et al., 2011; Plassard et al., 2011) and so meliorate their nutritional state (Gafur et al., 2004). Moreover, resource partitioning of P associated with mycorrhizal fungi could contribute to more efficient use of different P forms by plants, reducing competition for soil P (Turner, 2008). It is hypothesised that mycorrhizal fungi are the key component in resource partitioning under P impoverished conditions in soil.

Ectomycorrhizae (ECM) were shown to mine different chemical forms of P (reviewed by Plassard et al., 2011) in exchange for energy derived from hosts’ photosynthesis (Buscot, 2015) and increase cost-efficiently the soil volume explored for nutrients *via* its extra-radical hyphae (Jones et al., 1998). To understand if (i) ECM mobilizes P from differently accessible sources and (ii) consequences of higher energy costs for the plant development due to the acquisition of more complex P forms exist, adequate and innovative experimental systems consisting of compatible plants and mycorrhizal fungi have to be developed. The use of compatible plant and ectomycorrhizal (ECM) fungal species provide valuable model systems for a more robust test of nutrient acquisition and exchange models (Gafur et al., 2004). In functional symbiosis, the mycorrhizal associates can penetrate the plant root, forming a Hartig net between the cortical cells (Rousseau et al., 1994; Hampp et al., 1996; Gafur et al., 2004), where the exchange of nutrients between the symbionts is supposed to happen (Landeweert et al., 2001). In contrast, incompetent ECM fail to penetrate the host roots, causing a defence reaction by thickening the cell wall of the epidermis (Lei et al., 1990; Gafur et al., 2004). It was shown that only functional associates could increase plant P uptake in nature under impoverished nutrient conditions (Rousseau et al., 1994; Smith and Read, 2008; Hoeksema et al., 2010). Poplar plant species *Populus x canescens* and the ECM fungus *Paxillus involutus* strain MAJ are the reassuring organisms on this matter (Gafur et al., 2004; Müller et al., 2013).

Under field conditions, poplars can form symbiotic associations with different mycorrhizal types such as arbuscular mycorrhizal (AM) and ECM fungi (Khasa et al., 2002; Gherghel et al., 2014). The study of Gherghel et al. (2014) could show that *P. involutus* subsequent to *Rhizophagus irregularis* colonized poplar clones under various field conditions. Among poplars, *P. involutus* (Basidiomycetes) has a wide variety of hosts able to form ECM with many forest tree species belonging to gymnosperms and angiosperms (Duddridge, 1987; Baum et al., 2000; Gafur et al., 2004) and be an appropriate help for trees in ‘bare-root’ conditions (Jarosch and Bresinsky, 1999; Hönig et al., 2000). *P. involutus* was also shown to be able to exude oxalic acid to acquire P from mineral sources (Lapeyrie et al., 1991) and to release surface-bound phosphatases that can mineralize organic P forms (McElhinney and Mitchell, 1993; Alvarez et al., 2004). Moreover, *P. involutus* is easy to maintain and propagate in culture and is therefore increasingly used in ECM studies (Wallander and Soderstrom, 1999; Gafur et al., 2004; Müller et al., 2013).

The natural hybrid *P. x canescens* (grey poplar) result through pollination of *P. alba* (white poplar) by *P. tremula* (European aspen) (Lexer et al., 2005). *P. x canescens* and *P. alba* occur sympatrically in European river valleys (Rajora and Dancik, 1992; Fossati et al., 2004; Lexer et al., 2005), whereas *P. tremula* is an important pioneer tree species covering forests in the upland (Adler et al., 1994; van Loo et al., 2008). Furthermore, the economic and scientific importance of *Populus* trees increased. Due to their natural distribution and genetic variability, they can be cultivated under polluted and degraded soil conditions (Chen and Polle, 2010) and contribute to a site’s positive carbon balance. Thereby, the poplar supplies the industry with wood biomass, fibre, bioenergy and chemicals (Klass, 1998). Furthermore, poplars are considered as beneficial model organisms due to the ease of micropropagation (Confalonieri et al., 2003), which reduces stochastic variation by the use of clones instead of seedlings. Because of all these benefits, poplars gained importance in scientific fields, including biotechnology, molecular biology, and other areas related to nutrition, abiotic pressures, or the plant-soil interface (reviewed by Müller et al., 2013).

The latest and first study aiming to investigate the ECM mediated resource partitioning for P was performed by Schreider et al. (2022), revealing that the ECM *P. involutus* has the potential to occupy fundamental niches of various P sources, whereby the readily available phosphate was not as expected the most favourable P source for uptake within a mixed P pool. Previously, progress in studying partitioning for soil P has been achieved by examining plant responses to single P sources (Steidinger et al., 2014) or a mixed pool of different P sources (Liu et al., 2018), but the sole contribution of mycorrhizal fungi in resource partitioning was neglected in these studies. The studies of Andrino et al. (2019; Andrino et al., 2021) were the first to investigate the mycorrhizal mediated acquisition of differently available P sources using arbuscular mycorrhiza. Nevertheless, Andrino et al. (2019; Andrino et al., 2021) have determined that higher amounts of C were invested by the plant into the association with a mycorrhizal fungus that had access to more complex P sources, whereby the P sources were supplied as a single P source. By using separate compartments for the nutrients, it is possible to mimic the ecosystem situation with an mycorrhizal plant having access to widely distributed nutrient patches with different bioavailabilities through mycorrhizal fungus, excluding the direct competition of plant roots and mycorrhizal hyphae for P and depending on the experimental conditions revealing the true capabilities of mycorrhizal fungal associate in nutrient acquisition.

The main aim of this study is to provide practical experience and to describe the learning curve in developing a culture system that is robust and versatile to investigate mycorrhizal mediated resource partitioning. Three different experimental setups using poplar plantlets of *P. x canescens* and its functional ECM fungus *P. involututs* strain MAJ for axenic and greenhouse conditions are presented. The present study covers also modified protocols (Müller et al., 2013) of the *in vitro* multiplication and rooting of plant material and production of ECM fungal inoculum under axenic conditions that suited our experiments the most. Furthermore, the co-cultivation of poplar and ECM fungi and maintenance steps in different culture systems under axenic and greenhouse conditions are reported. Also, the construction of separate compartments for the nutrient supply that are adaptable into different experimental set-ups is described.

## Axenic Multiplication and Rooting of Plant Material

All steps regarding multiplication, maintenance, and preparation of plant and fungal material were performed under sterile conditions under laminar airflow. As a model plant, poplar species are beneficial because of their ease for *in vitro* propagation (Müller et al., 2013). For the propagation of poplar plantlets *P. x canescens* clone ‘Schleswig I,’ Woody Plant Medium [WPM, (Lloyd and McCown, 1980) composed as published by Müller et al. (2013)] without hormones (ready to use purchased from dephyte e.K., Langenberg, Germany) was used. After pH was adjusted to 5.2, 8 g/L plant agar (Duchefa Biochemie B.V, Haarlem, Netherlands) was added as a solidifying agent. Explants were established from lateral meristems with one leaf and bud. Always eight cuttings were placed in one vessel (sterile, PP, 10 cm high, 7 to 9 cm in diameter; Plastikbecher.de GmbH, Giengen an der Brenz, Germany) containing around 70 ml WPM multiplication medium (autoclaved at 121°C for 20 min) and these vessels kept under controlled environmental conditions (24°C; 60% humidity; photosynthetic active radiation (PAR) of 150 µmol m^-2^ s^-1^ at plant height for a light period of 16 h). The multiplication step was repeated every eight weeks to maintain the culture homogeneous and fresh. This period of time has to be considered if high quantities of plantlets for the experiments have to be produced starting with a few vessels only.

For rooting, the poplar plantlets’ apical buds with 1.5 cm height were used. The WPM medium used for rooting contained one-third of sucrose and vitamins only. The medium was poured (around 90 ml) into Microboxes (autoclavable, 13 cm high, 9 to 10 cm in diameter; O118/80, by Sac O_2_, Deinze, Belgium). The leads of these Microboxes had an installed membrane for sterile gas exchange (81.35 GE day^-1^) so that CO_2_ could diffuse into and O_2_ outside the vessel. The last two steps were necessary to shorten the acclimatization time of the plants in the greenhouse so that the plants already start to perform photosynthesis, not only taking C from the medium. After six weeks, the plantlets developed roots of up to 8 cm in length and were 6 to 8 cm in height. In this developmental stage, the plantlets are ready to be transferred into the culture systems and the direct co-culture with the mycorrhizal fungus.

## Axenic Production of Ectomycorrhizal Inoculum

Before used as inoculum, the ectomycorrhizal fungus *P. involutus* strain MAJ was propagated and maintained in pure culture on modified Melin-Norkrans medium (MMN, ready to use salts purchased from dephyte e.K., Langenberg, Germany). The composition of MMN medium was published by Müller et al. (2013). After the pH of the medium was adjusted to 5.5, 10 g/L plant agar was added as a solidifying agent. Petri dishes (ø 9 cm) were filled with 25 ml MMN medium (autoclaved at 121°C for 20 min) and inoculated by a fungal plug from the previous culture. Petri dishes were sealed with Parafilm, wrapped in aluminium foil to simulate darkness, and kept with the lid down at room temperature at around 20 to 23°C.

For different culture systems, there exist various ways to produce the inoculum of *P. involutus*. The cultivation techniques of ECM fungi were reviewed in detail, e.g. by Harvey (1991). For our experimental purposes following techniques worked well: For *in vitro* systems, it is suggested to pre-grow the ECM fungus on a cellophane membrane (CM; Gel drying frames, Sigma, Z377600-1PAK, LOT# 3110) in axenic culture (Felten et al., 2009; Navarro-Rodenas et al., 2012; Müller et al., 2013). The CM would stop the fungus from growing into the medium and, due to its fine porosity, it would supply the fungus with the necessary nutrients from the medium (Müller et al., 2013). The CM would ease the transfer of the fungus into the culture system that is maintained on a different medium that is different from the propagation medium and should not contain specific elements (e.g. P, C, or N) and vitamins. Therefore, to prevent this, the fungal plug should be cut out before using the pre-inoculated CM for the experiments. Further benefits of using CM is to shorten the time for the mycorrhization of the plant and to foster the proliferation of mycelium in the system. The CM (1.6x6cm) was boiled in 1 mM EDTA (Merck, 1.08417.1000) solution for one hour and washed in deionized water (dH_2_O) (Navarro-Rodenas et al., 2012). After autoclaving, the CM was plated on MMN medium, inoculated with two fungal plugs of MAJ, and sealed and incubated as described above.

In experiments with mesocosms or rhizotrone systems which use growth substrates like Perlite, it is necessary to produce higher quantities of inoculum, as it is usually applied proportions of around 10% compared to the substrate [reviewed by Harvey (1991)]. This is possible by propagating *P. involutus* in liquid culture. For this purpose, one culture plate of *P. involutus* has to be vortexed and transferred in 0.2 L MMN liquid medium in 0.5 L glass bottles. Under horizontal shaking for around eight weeks, the fungus grows in more or less concentric spheres, which can be perfectly used to proliferate further on 0.8 L Perlite carrier and 0.2 L MMN. The later step is required to obtain a more homogeneously inoculated substrate for the actual experiments.

## Co-Cultivation in Compartmental Culture Systems

**Figure 1:**
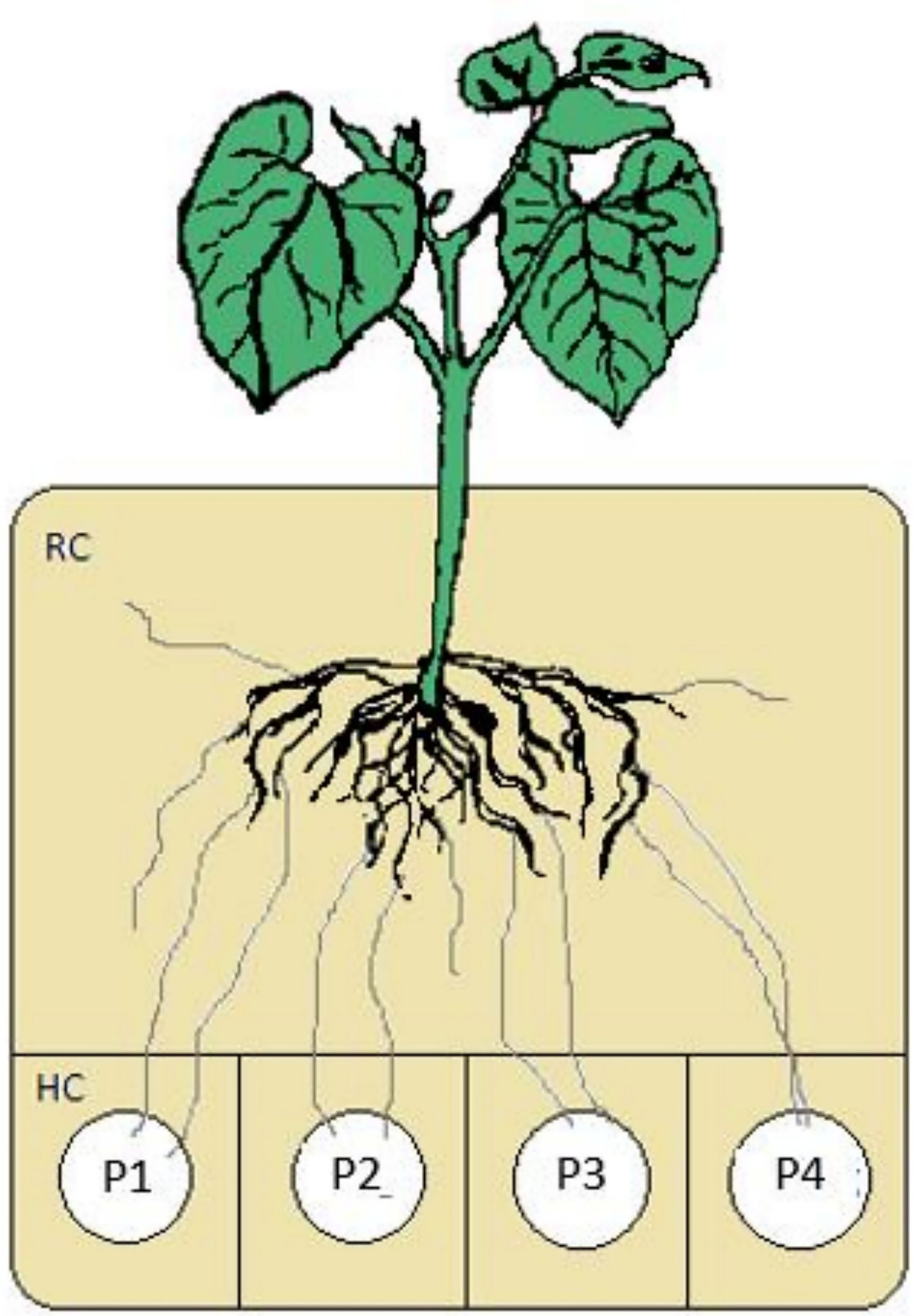
Experimental setup for mycorrhizal mediated uptake of P of different bioavailabilities and translocation to the plant.

In the following study, the presented culture systems consisted of a root compartment (RC) and physically separated hyphal compartment (HC) to exclude the plant roots from the contents of the hyphal compartments (**Figure 1**). The design of the systems allows the insertion of more than one HC. For our aims, up to four HCs with different chemical forms of P, representing different bio-availabilities, were introduced into the RC.

Before deciding about the experimental setup, the designer has to consider the key boundary conditions: (i) the aim/research question, (ii) the variables and parameters that has to be measured or visualised, (iii) the experimental necessities to test the hypothesis (e.g. axenic or outdoor conditions), or (iv) the resources to establish the system. The following presents three different experimental approaches with their benefits and disadvantages.

### Indication of the Hartig Net in Mycorrhizal Roots of *P. x canscens* Associated With *P. involutus*

**Figure 2:**
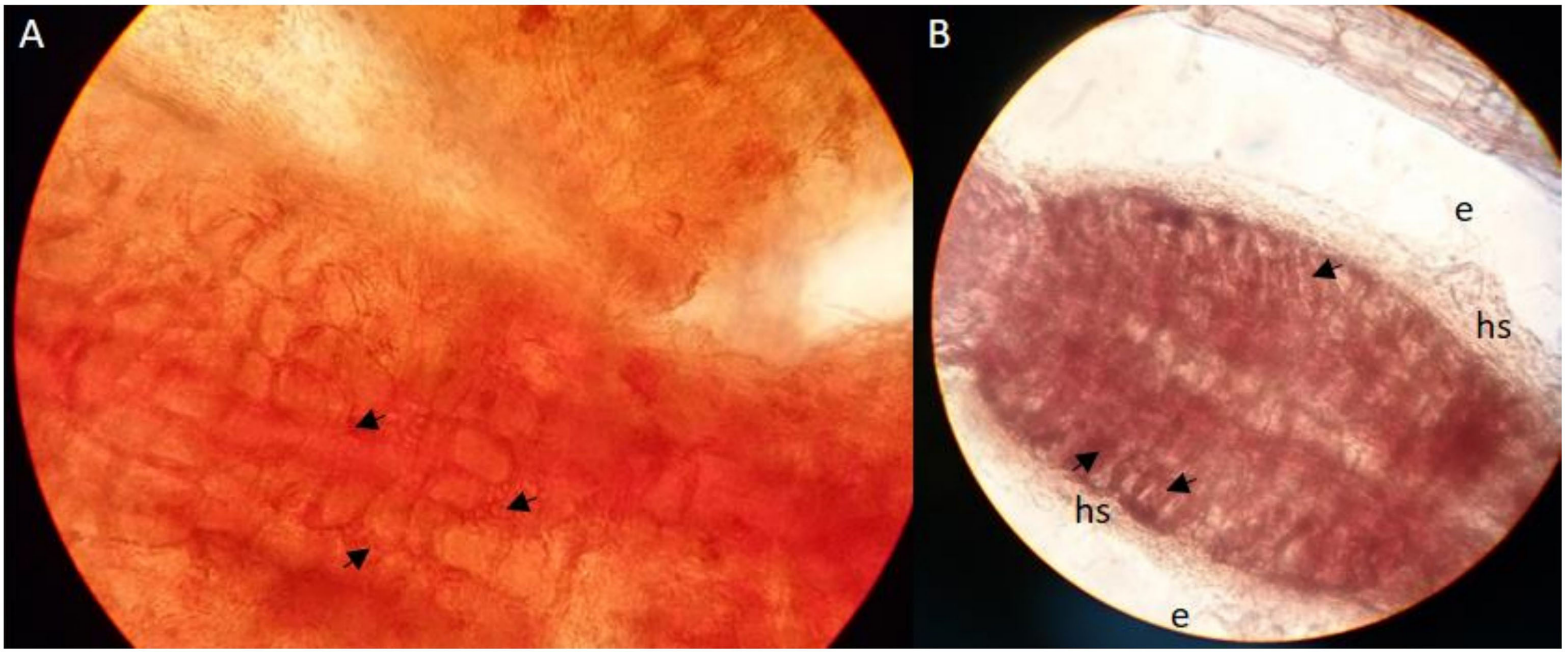
Cleared and stained (acid fuchsin) root tips [**(A)** x100 magnification and **(B)** x40 magnification] of * P. x canescens * (Schleswig I) colonised by ECM fungus *P. involutus* (MAJ). The arrows indicate the Hartig net hyphae between the root cortical; hs: hyphal mantle around the root tips (mycorrhizal root tips).

Characteristically for successful mycorrhization of poplar roots by MAJ is the change in root morphology such as specific branching of root tips and absent development of root hairs (Brundrett et al., 1996). From our pre-experiment of synthesizing *P. x canescens* with *P. involutus*, we could observe the Hartig net hyphae between the cortical cells inside the roots (**Figure 2**) and a dense hyphal mantel around the root tips (**Figure 2B**). Such observations of *P. x canescens* with *P. involutus* (MAJ) were also previously detected by Gafur et al. (2004).

### Phosphorus Sources and Their Sterilisation

In our studies, we have tested resource partitioning for soil P using the following P chemical forms to mimic the conditions and cover the most abundant P pools available in soil: *ortho*-phosphate (oP), organic P source [P_org_; e.g. Phytate (Phy) or adenosine monophosphate (AMP)], mineral P source (e.g. apatite (AP; Krantz Company, Bonn, Germany) or synthesised hydroxyapatite [HAP; according to Wolff et al. (2018)]; < 0.2 mm), oP secondary mineral adsorption complex (oP bound to goethite [gP; according to Andrino et al. (2019)]; < 0.63 mm). The sources oP and P_org_ were supplied in liquid form and filter-sterilised using a sterile syringe filter (Filtropur S, PES, 0.2 µm pore size, Sarstedt AG & Co. KG, Nümbrecht, Germany) prior filling into the HCs. In contrast, the mineral sources underwent a fractionated sterilisation (three times sintered at 105°C for 30 min over three days), which is called tyndallization and is used when the mineral structure should not be changed through the sterilization process. The consistency of gP with a high volume to mass ratio compared to other P sources has to be taken into account when considering the amounts of each P source that have to be supplied to the system at the HC.

### *In vitro* Culture System With Controlled Conditions

Successful *in vitro* synthesis of plants of *Populus* sp. and ectomycorrhizal fungi, including *P. involutus*, were reported in previously conducted studies (e.g. Hampp et al., 1996; Gafur et al., 2004; Felten et al., 2009; Müller et al., 2013). First attempts were to use divided Petri dishes in order to create a compartmental *in vitro* system for studying AM uptake of P (Joner and Johansen, 2000; Koide and Kabir, 2000; Nielsen et al., 2002) and other elements (Dupre de Boulois et al., 2006; Dupre de Boulois et al., 2009). We have combined these practices to create a compartmental *in vitro* system to study resource partitioning and elemental exchanges between the mycorrhizal fungus and its host plant.

**Figure 3:**
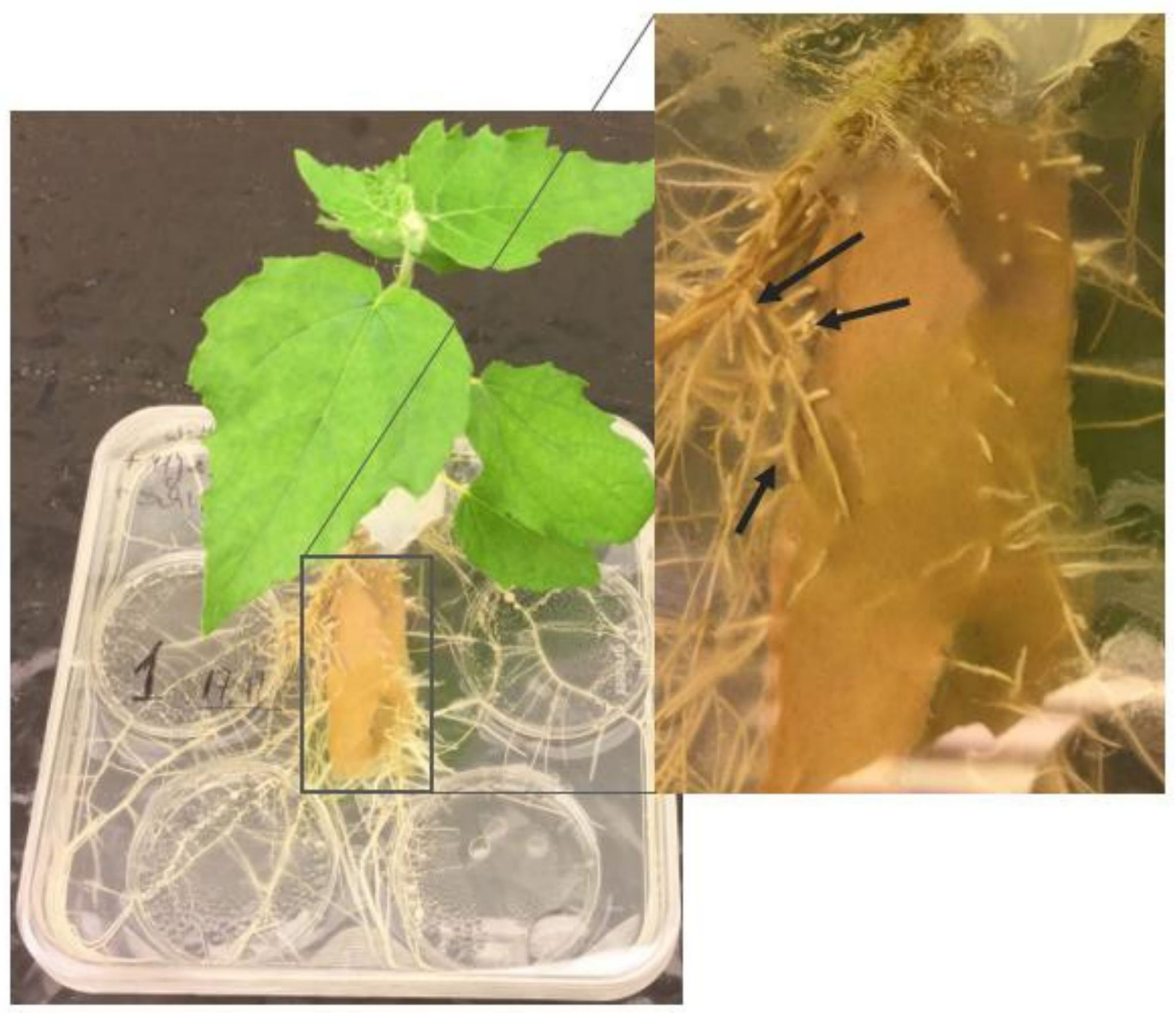
Compartmental *in vitro* culture system synthesising *P. x canescens* (Schleswig I) and the ectomycorrhizal fungus *P. involutus* (MAJ, pregrown on CM) on WPM medium lacking P, C, and vitamins; at 17 dpi and maintained at 24°C and >80% humidity. The arrows in the zoom-in point to specific branching of fine root tips that indicate successful mycorrhization of plant roots.

All steps were conducted under sterile conditions under laminar airflow. The culture system (**Figure 3**) consisted of a square Petri dish (120 x 120 x 10 mm, sterile; Greiner Bio-One GmbH, Frickenhausen, Germany), serving as RC. The MMN media formulation was shown to be too poor for successful co-cultivation of *P. tremula *L. and *P. involutus* (Langer et al., 2008). After macronutrients and vitamins were introduced into the formulation, the plant survival rate and mycorrhization increased. The WPM medium formulation contains all necessary macro- and micronutrients for plant development and, from our own experience, also for a successful mycorrhization. Therefore, Petri dishes were poured with around 70 ml WPM medium lacking P, C, and vitamins. As long as the medium was warm, four lids of Petri dishes (ø 35 x 100 mm, sterile; Sarstedt AG & Co. KG, Nümbrecht, Germany), serving as HC, were pressed slightly into the medium so that the medium extended bellied above the lid wall (Dupre de Boulois et al., 2006). Due to the gravitational attraction, the plant roots could grow into the medium underneath the HCs. The HCs were filled with four different, sterile P sources. The CM pre-inoculated with *P. involutus* was placed on the top of the medium between the HCs and the plant with the roots on the top of the CM to obtain mycorrhizal treatments or directly on the top of the medium to obtain non-mycorrhizal controls. The RC was closed with the lid of the Petri dish, where on one side or on the top, a hole was provided for the plant shoot and sealed with a sterile (autoclaved at 121°C for 20 min) silicon grease (Voets et al., 2005) to avoid contamination. The RC of the system was sealed with Parafilm and wrapped in aluminium foil to maintain the roots in the dark. A successful mycorrhization (**Figure 3**) of *P. x canescens* and *P. involutus* was clearly detectable after 17 days post-inoculation (dpi).

**Figure 4:**
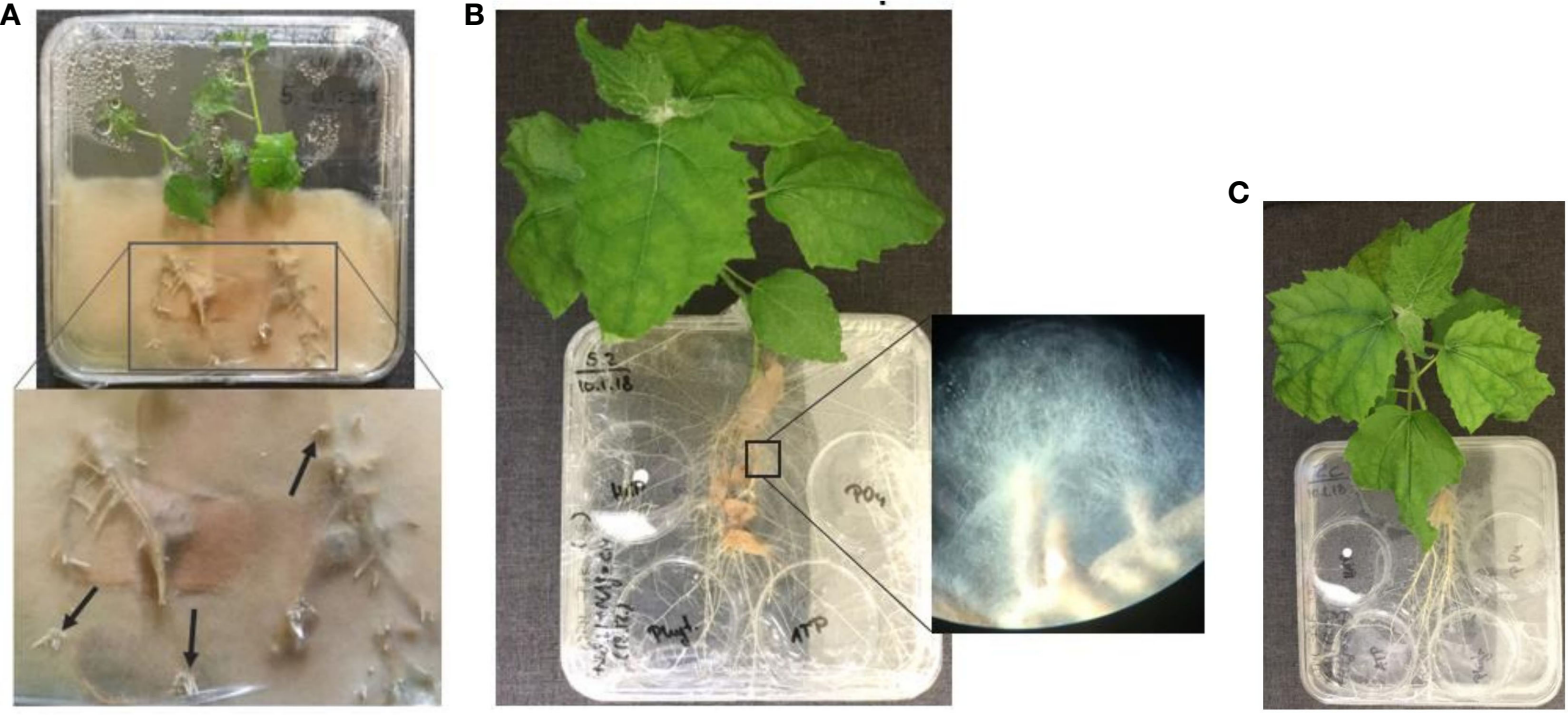
**(A)** Pre-inoculated *P. x canescens* (Schleswig I) by the ectomycorrhizal fungus *P. involutus* (MAJ, pre-grown on CM) in a closed culture system on WPM medium containing one-third P, C and no vitamins (22 dpi); **(B)** Compartmental *in vitro* culture system prepared from pre-inoculated Schleswig I with MAJ (pregrown on CM) and **(C)** the non-mycorrhizal control poplar plant on WPM medium lacking P, C, and vitamins (55 dpi). All systems were maintained at 24°C and >80% humidity. The arrows in the zoom-in **(A)** point to specific branching of fine roots tips that indicate successful mycorrhization of plant roots; and **(B)** show the proliferation of fungal mycelium from the mycorrhizal root tips in the culture medium.

To shorten the time for mycorrhization of plant roots, the plant can be pre-inoculated in a closed culture system (**Figure 4A**). The same square Petri dishes were used. Only a half of the petri dish was filled with WPM media, containing only one-third of P and C as required from the formulation and no vitamins. Two plants were placed per system with roots on top of CM pre-inoculated with *P. involutus* and the shoots inside the dish. These systems were sealed with Parafilm, and the bottom part was covered in an opaque plastic bag to keep the roots in the dark. After 22 dpi, the plants can be transferred into the compartmental *in vitro* systems (**Figure 4B, C**). We could see a proliferation of up to 2 cm of fungal mycelium from the mycorrhizal root tips in the medium after only 33 dpi (**Figure 4B**).

These systems are vulnerable to desiccation and thus have to be maintained under high humidity > 80%, a temperature of around 22°C, and PAR of 200 µmol m^-2^ s^-1^. In the first two weeks, the freshly transferred plantlets in the system were covered with translucent vessels (e.g. sterile PP vessels used for plant material propagation). Especially, the non-mycorrhizal control plants were sensitive to desiccation and started to wilt after around 80 dpi, indicating that the ECM fungus *P. involutus* makes the plant more resilient to abiotic stress. If the high humidity in the growth chamber cannot be preserved, it is possible to keep the systems in a translucent container that can be closed. But they need to be opened regularly to ensure an exchange of CO_2_ and O_2_. Further, the *in vitro* systems are prone to contamination. With each additional preparation step, the contamination rate of the prepared systems increases. To maintain the system, new medium has to be added frequently to the system after around 80 dpi. For that the system has to be opened, increasing the contamination rate.

Besides the complicated maintenance, the *in vitro* culture system has also benefits: By using this type of culture system with agar or gellan gum as a solidifying agent for the medium in RC, the roots and fungal structures can be extracted from the gel using sodium citrate buffer [e.g. 10 mM, pH 6.6, at temperatures above 30°C (Doner and Becard, 1991)]. This makes the mycorrhizal fungal mycelium and plant roots well accessible for various analyses. The plant roots can easily be separated, and the hyphae filtered from the medium in the RC. This culture system also enables the collection of hyphae from the HCs separately by cutting them at the RC-HC interface.

Nevertheless, the proliferation of fungal mycelium in substrates like Perlite is faster. Therefore, one should consider whether it is necessary to make all this effort to establish an *in vitro* culture system or focus on other culture systems, which are more robust and much easier to maintain.

### Preparing Separate Hyphal Compartments for Axenic Rhizotrone and Mesocosm Culture Systems

The separate hyphal compartments (HCs) for the P sources can be easily prepared from different SafeSeal reaction tubes to Falcon tubes to plastic bottles, depending on the experimental scale. For our culture systems, the HCs were prepared of (a.) 5 ml SafeSeal reaction tubes or (b.) 15 ml Falcon tubes (**Supplementary Figure 1**) (both from Sarstedt AG & Co. KG, Nümbrecht, Germany). For construction of both, at around 2 ml height a hole of 1 cm in diameter was burned into the tube and sealed with a combination of two types of membranes. A hydrophobic PTFE membrane (5 µm, Pieper Filter GmbH, Bad Zwischenahn, Germany) was installed at the P source side, preventing the liquids from mass flow and ions from diffusion into the root compartment (Mäder et al., 1993; Vierheilig et al., 1998; Andrino et al., 2019). A nylon mesh (20 µm, Franz Eckert GmbH, Waldkirch, Germany) was attached on the top of the PTFE membrane at the plant root side (Watkins et al., 1996), protecting the hydrophobic PTFE membrane additionally from a higher force applied by a growing root. The HCs were autoclaved at 121°C for 20 min and filled with the different P sources under sterile conditions. All containers were filled up to (a.) 3 ml or (b.) 4 ml with dH_2_O. The HCs filled with P sources were always prepared at least one day before using them in the culture systems. If the HCs were prepared for axenic culture systems, they were stored in sterile containers until use.

### Axenic Rhizotrone Culture System With Controlled Conditions

With the axenic rhizotrone culture systems (**Supplementary Figure 2**), we have excluded the weaker points of *in vitro* culture system to establish a more robust compartmental culture system with an additional feature to supply the plants in the culture systems with the nutrient solution without the need to open it. This system also offers controlled conditions in the belowground in relation to rhizo- and mycosphere.

All preparing steps were conducted under sterile conditions under laminar airflow. The rhizotrone culture systems (**Supplementary Figure 2**) were made of square Petri dishes (100 x 100 x 20 mm, sterile; Sarstedt AG & Co. KG, Nümbrecht, Germany). The HCs used for this culture system were made of 5 ml SafeSeal reaction tubes, and up to four of these HCs can fit into the system. Four HCs were filled with different chemical forms of P such as oP, AMP, HAP, and gP. In a randomized order, these HCs were inserted one centimetre from the bottom into the rizotrones. Then, the rhizotrones were filled with Perlite (Perligran^®^ classic, Knauf Aquapanel GmbH, Dortmund, Germany) as a nutrient free substrate. The Perlite was two times washed with dH_2_O and autoclaved (at 121°C for 20 min). To obtain treatments with mycorrhized plants, the substrate was inoculated with 10% of *P. involutus* that was proliferated on Perlite as a carrier. For the non-mycorrhizal plant treatments, the inoculated substrate was autoclaved one more time before use. The rooted poplar plantlets were placed with the roots on the top of the HCs and covered with an additional thin layer of Perlite. The rhizotrone can be covered with the lid of the Petri dish. But for our experiments, we have used a thin sterile PVC foil (100 x 100 x 0.2 mm, Modulor GmbH, Germany) as a cover glued with a hot glue. Compared with the original lid from the Petri dish, the foil reduces the shielding of radio-isotopes (e.g. ^33^P). The skip for the plant stem at the top and the opening to release the excess of nutrient solution at 2 cm height from the bottom of the rhizotrone were closed with a sterile cotton plug.

To maintain axenic conditions of the system during watering, the rhizotrones were equipped with a sterile syringe filter (Filtropur S, PES, 0.2 µm pore size, Sarstedt AG & Co. KG, Nümbrecht, Germany), which was connected to the rhizotrone through an autoclaved PVC pipe. From 0 dpi, the mesocosms were supplied with a WPM nutrient solution containing macro- and micro-elements without P and vitamins (WPM –P), which was balanced with KNO_3_ and (NH_4_)_2_SO_4_ to adjust the desired concentration of K. This is a modified formulation described by Müller et al. (2013). WPM nutrient solution were purchased as ready-to-use salts (dephyte e.K., Langenberg, Germany) that had to be dissolved in dH_2_O only.

To obtain no P controls, the rhizotrones were prepared with HCs containing dH_2_O only. The plantlets were kept under a plastic cover and included moisturized protecting paper plugs for the first two weeks for acclimatization and, thus, for protection from lower ambient air humidity and higher UV radiation. The rhizotrones were kept in a climate chamber at 80% ambient humidity and 20/18°C at an 18 h/6 h day/night cycle.

The results and the discussion of this experiment are published in Schreider et al. (2022).

### Mesocosm Culture System With Greenhouse Conditions

**Figure 5:**
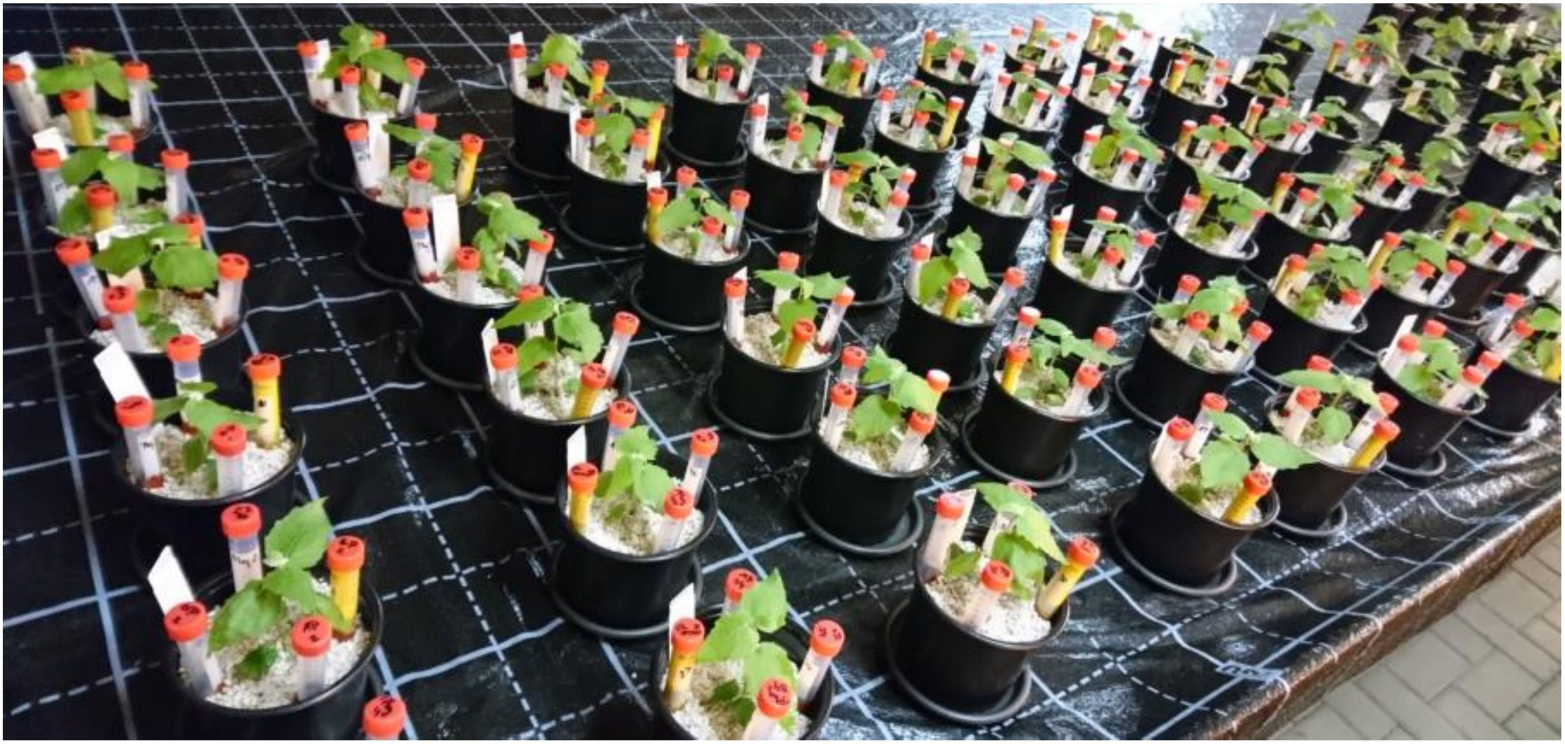
Mesocosm culture systems with four different chemical forms of P (4P) supplied in four separate HCs under greenhouse conditions: the poplar plants *P. x canescens* (Schleswig I) mycorrhized by ectomycorrhizal fungus *P. involutus* (MAJ) and non-mycorrhizal control plants at 58 dpi. The HCs for the different P sources were made of 15 ml Falcon tubes and hydrophobic and nylon meshes (see **Supplementary Figure 1** and **Figure 7**).

If the controlled (axenic) conditions are not a key boundary for the research aim, the compartmental system can be up-scaled to the greenhouse conditions. In this paragraph we describe a mesocosm culture system (**Figure 5**) to investigate mycorrhizal mediated resource partitioning for P under greenhouse conditions.

The mesocosm culture systems (**Figure 5**) were prepared from pots and HCs (**Supplementary Figure 1**) to supply the different P sources. The rooted poplar plantlets of *P. x canscens* were transferred directly in pots (Ø 13 cm, 0.83 L) with Perlite as substrate (washed with dH_2_O and autoclaved at 121°C for 20 min two times). The substrate was inoculated with 10% of *P. involutus* (proliferated on Perlite as a carrier) to obtain mycorrhizal plant treatments. For the non-mycorrhizal plant treatments, the inoculated substrate was autoclaved one more time before use. The mycorrhizal and non-mycorrhizal plantlets were kept under a plastic cover and a protecting cloth for four weeks for acclimatization, as the temperature in December in the greenhouse could drop to 14°C. In other seasons, the acclimatization period can be shortened to two weeks. The mesocosms were supplied with a WPM nutrient solution (without vitamins) containing a low concentration in P of 1.1 mg l^-1^ (WPM LP) as described by Müller et al. (2013) to support the P nutrition before the mycorrhizal fungus starts to acquire P from the HCs. At 58 dpi, four separate HCs containing each a different chemical P form such as oP, Phy, AP, or gP were inserted into the mesocosms (**Figure 5**). From 72 dpi, the mesocosms were watered with a WPM solution without any P (WPM –P). Both nutrient solutions WPM LP and WPM –P were balanced with KNO_3_ and (NH_4_)_2_SO_4_ to adjust the concentration of K. All WPM nutrient solution variants were purchased as ready-to-use salts (dephyte e.K., Langenberg, Germany). In the experiment’s time course, the mesocosms were watered with dH_2_O on demand.

**Figure 6:**
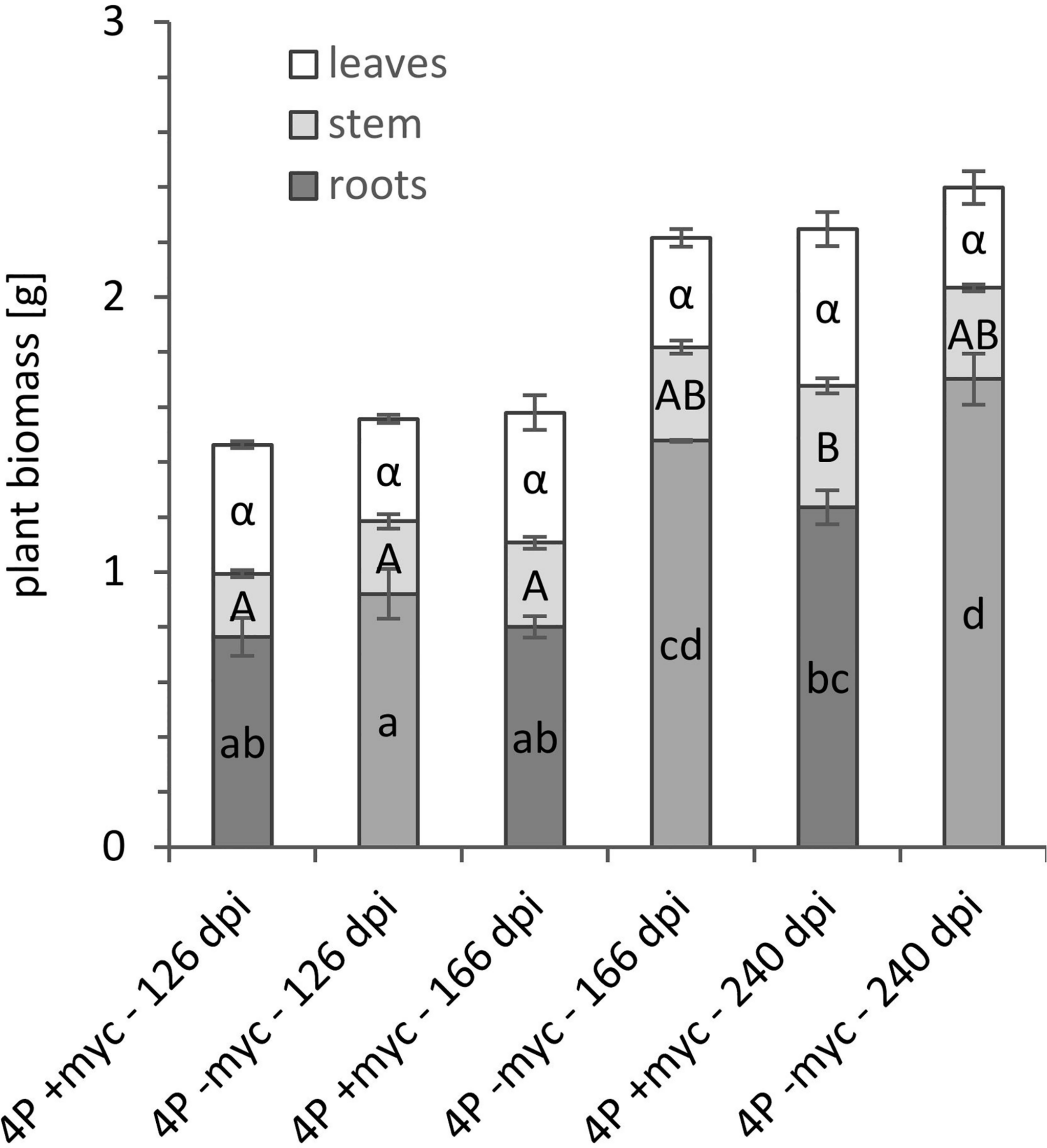
Plant biomass [mg] in *P. involutus* ectomycorrhizal (+myc) and non-mycorrhizal (-myc) poplar plants of *P. x canescens* from 4P treatments harvested at 126, 166, and 240 dpi. The error bars show the standard deviation (n=3). The letters in the bars indicate significant differences in plant biomass [g] between mycorrhizal treatments and harvesting time points: *P* < 0.05.

**Figure 7:**
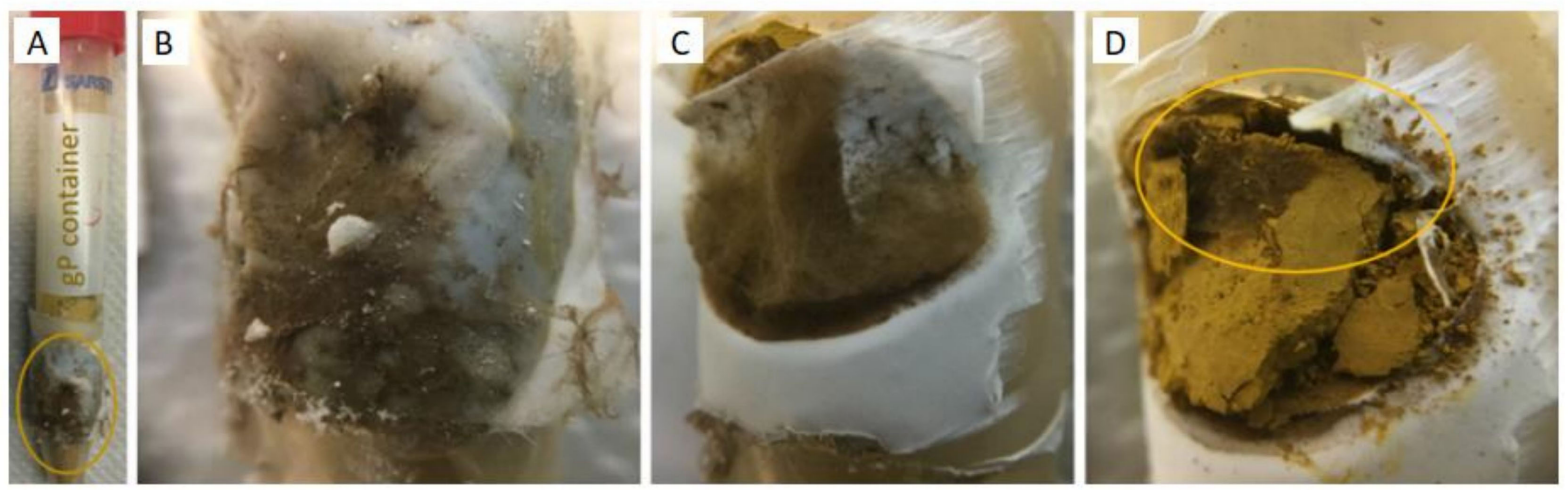
**(A)** HC containing gP as a P source at 166 dpi; **(B)** nylon mesh, **(C)** recumbent hydrophobic PTFE membrane, and **(D)** gP colonised by *P. involutus* (MAJ).

The poplar plantlets and *P. involutus* were co-cultured in the mesocosm culture system for up to eight months. In case the experimental period has to be longer than ten months, we suggest using bigger pots, as the non-mycorrhizal plants respond to P deficiency with higher root biomass (**Figure 6**) in foraging for P. Also, in this culture system, we could observe abundant branching of mycorrhizal fine root tips specific for *P. involutus* (**Supplementary Figures 3**, **4**), confirming high mycorrhization of poplar roots. The substrate and, especially, the entrance points to the P sources at HCs (**Supplementary Figure 3** and **Figures 7A, B**) were highly colonised by the mycelium of *P. involutus*. We could also observe the mycorrhizal mycelium between the membranes (**Figures 7C**) and inside the HC (**Figure 7D**) of all provided P sources in mycorrhizal treatments.

**Figure 8:**
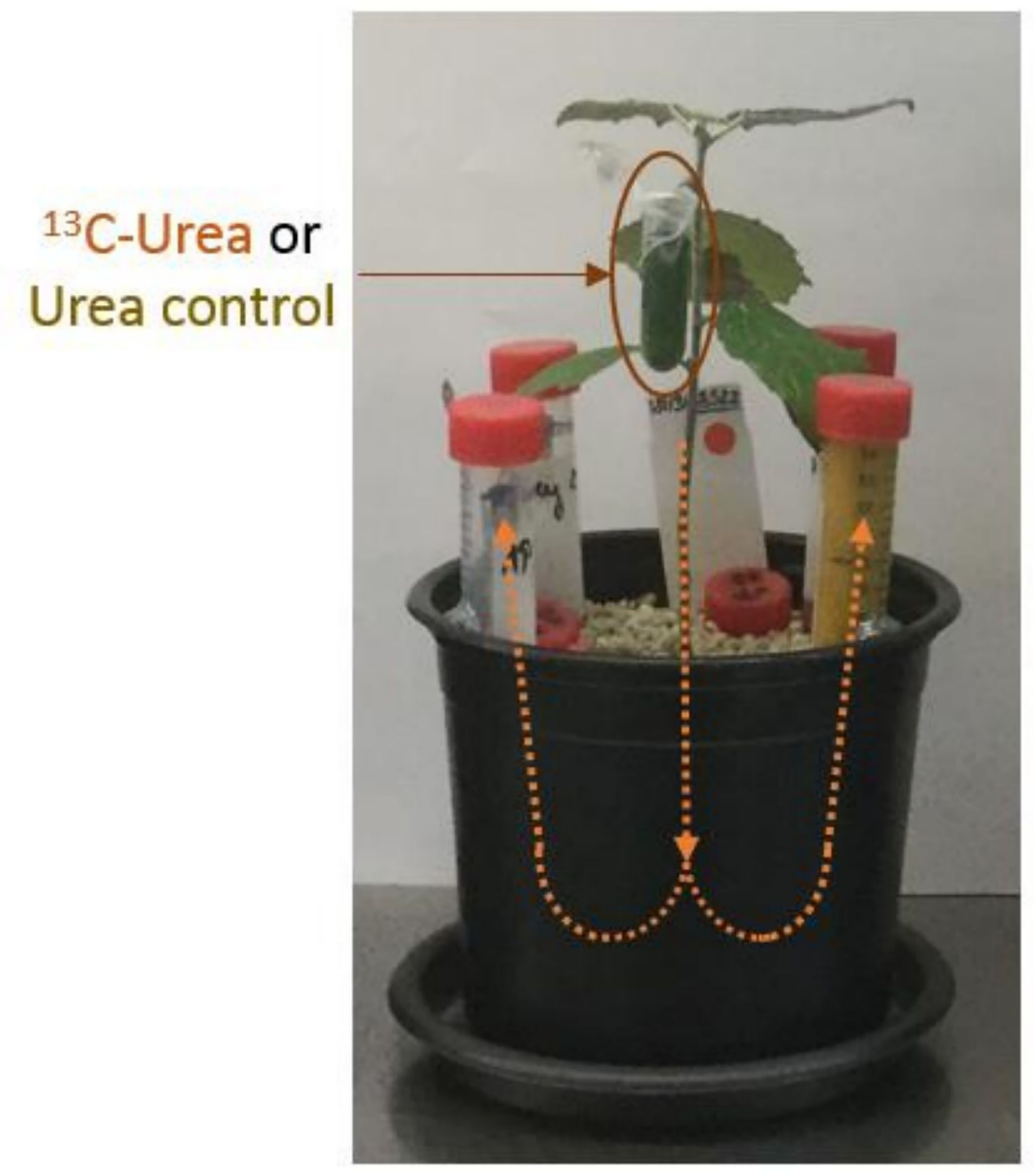
^13^C-labelling of *P. involutus* ectomycorrhizal poplar plant *P. x canescens* using leaf-fertilisation via ^13^C-labelled urea or non-labelled urea as controls at 167 dpi.

Compared to the axenic culture systems, the mesocosms are more robust after a short acclimatization period, easy to maintain and construct, and very versatile. Depending on the number and amount of supplied resources with the HCs and further additional tests, the system can be up- or down-scaled by using a different size of pots and containers used to prepare the HCs. This system can be easily used for labelling experiments, such as labelling the plant with ^13^C-Urea *via* leaf-fertilisation to trace the C transfer from plant to the mycorrhizal fungus growing towards the different P sources (**Figure 8**). Different transfer rates of labelled C can indicate different investment costs required from the mycorrhizal partner to acquire the different chemical forms of P (Andrino et al., 2019). Further tests could include studying the P acquiring mechanisms of the mycorrhizal fungus by measuring the enzymes or low molecular organic anions in the HCs containing the different P sources.

## Conclusion

The present study revealed that the construction and maintenance of the axenic rhizotrone and the mesocosm culture systems are less complicated and time consuming compared with the *in vitro* culture system. But also, the *in vitro* culture system can be equipped with the external apparatus with sterile syringe filters (as reported for the axenic rhizotrone culture system) to supply the plants with a nutrient solution without the need to open the system in the time course of the experiment. Nevertheless, especially the mesocosms are robust and very versatile. But all presented culture systems enable the user to comprise additional tests, including labelling of the plant with ^13^C or determination of P acquisition mechanisms.

Also the separate compartments for nutrient supply are well adaptable to different experimental set-ups and enable the simulation of an ecosystem situation with an ECM plant having access to widely distributed P source patches with different bioavailabilities (**Figure 1**) through mycorrhizal fungus, excluding the direct strife of plant roots and mycorrhizal hyphae for P. *P. involutus* is a long distance exploration type ECM fungus (Gronbach, 1988) with few but highly differentiated rhizomorphs (review by Agerer, 2001). These type of ECM fungi were shown to transport efficiently water and higher rates of P. Moreover, the ectomycorrhizal fungus *P. involutus* (strain MAJ) is compatible with the poplar plant species *P. x canescens* (clone ‘Schleswig I’) and both are easy to maintain and propagate *in vitro*. Hence, these organisms provide valuable model systems for a more robust test of nutrient acquisition and exchange models (Gafur et al., 2004; Müller et al., 2013).

Therefore, the design of a compartmental culture system using these compatible ectomycorrhizal associates was a solid choice to down-scale the ecosystem situation of P source dependent host C exchange for mycorrhizal P as well as of mycorrhizal mediated P resource partitioning. Since the protocol described by Rygiewicz et al., 1988, the present study was the first providing details on practical experience and evaluated protocols for the design and maintenance of the experimental set-ups to investigate such ecosystem situations. Moreover, these culture systems were designed not only for outdoor but also for controlled conditions excluding interferences with other micro-organisms, revealing the true capabilities of the mycorrhizal fungi in nutrient acquisition.

## Data Availability Statement

The original contributions presented in the study are included in the article/**Supplementary Material**. Further inquiries can be directed to the corresponding author.

## Author Contributions

The idea of the experiment came from JB with contributions from KS, AA, GG, AF, and LS. KS prepared the plant and fungal material, prepared the different culture systems, and conducted the experiments. KS performed data analysis and wrote the manuscript. JB and GG supervised the research. All authors contributed to the article and approved the submitted version.

## Funding

We want to thank the German Federal Ministry of Education and Research for the funding of this project in the framework of the DFG-RTG 1798 “Signaling at the Plant-Soil Interface”. The publication of this article was funded by the Open Access fund of Leibniz Universität Hannover.

## Conflict of Interest

The authors declare that the research was conducted in the absence of any commercial or financial relationships that could be construed as a potential conflict of interest.

## Publisher’s Note

All claims expressed in this article are solely those of the authors and do not necessarily represent those of their affiliated organizations, or those of the publisher, the editors and the reviewers. Any product that may be evaluated in this article, or claim that may be made by its manufacturer, is not guaranteed or endorsed by the publisher.

## Supplementary Material

Supplementary Figure 1 The separate hyphal compartments (HCs) for the different P sources made of 15 ml Falcon tubes and hydrophobic and nylon meshes.

Supplementary Figure 2 Axenic rhizotrone culture system with four different chemical forms of P (4P) supplied in four separate hyphal compartments (HCs): the poplar plants *Populus x canescence* (Schleswig I) **(A)** mycorrhized (+Myc) by ectomycorrhizal fungus *Paxillus involutus* (MAJ) and **(B)** non-mycorrhizal (-Myc) control plants at 7 dpi. The HCs for the different P sources were made of 5 ml SafeSeal tubes and hydrophobic and nylon meshes.

Supplementary Figure 3 Root tips of *Populus x canescence* (Schleswig I) colonised by the ectomycorrhizal fungus *Paxillus involutus* strain (MAJ) and its mycelium traversing the substrate, holding it as a block (at 126 dpi; viewed from **(A)** the side and **(B)** from above); hs: hyphal mantle around the root tips (mycorrhizal root tips); e: extraradical hyphae.

Supplementary Figure 4 Mycorrhization grade of plant roots expressed as mycorrhizal root tips per cm root length of *Paxillus involutus ectomycorrhizal* (+myc) roots of poplar plants *Populus x canescens* from different harvesting time points (126, 166, 250, and 243 dpi). The root length was determined using the grid-line intersection method (Giovannetti and Mosse, 1980; Brundrett et al. 1996). The error bars show the standard deviation (n=4). There was no significant (*P* < 0.05) difference in the mycorrhization grade of root tips between the different harvesting time points.
