# Supplementary figures and images for: Designing a Robust and Versatile System to Investigate Nutrient Exchange in, and Partitioning by, Mycorrhiza (Populus x canesces x Paxillus involutus) Under Axenic or Greenhouse Conditions
Source: Front Fungal Biol. 2022 Jun 17;3:907563. doi: 10.3389/ffunb.2022.907563 (PMC10512296; doi:10.3389/ffunb.2022.907563)

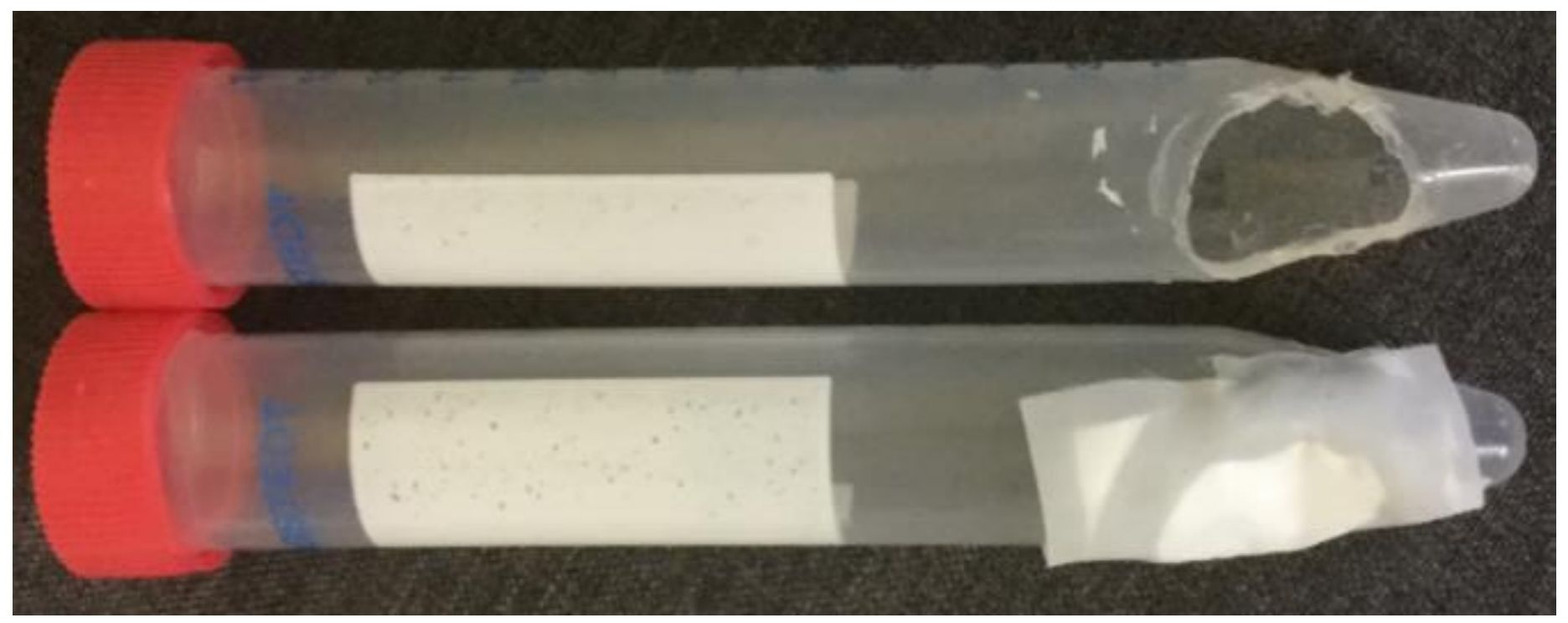

Supplement: Supplementary Figure 1 — The separate hyphal compartments (HCs) for the different P sources made of 15 ml Falcon tubes and hydrophobic and nylon meshes. [file Image_1.jpg]

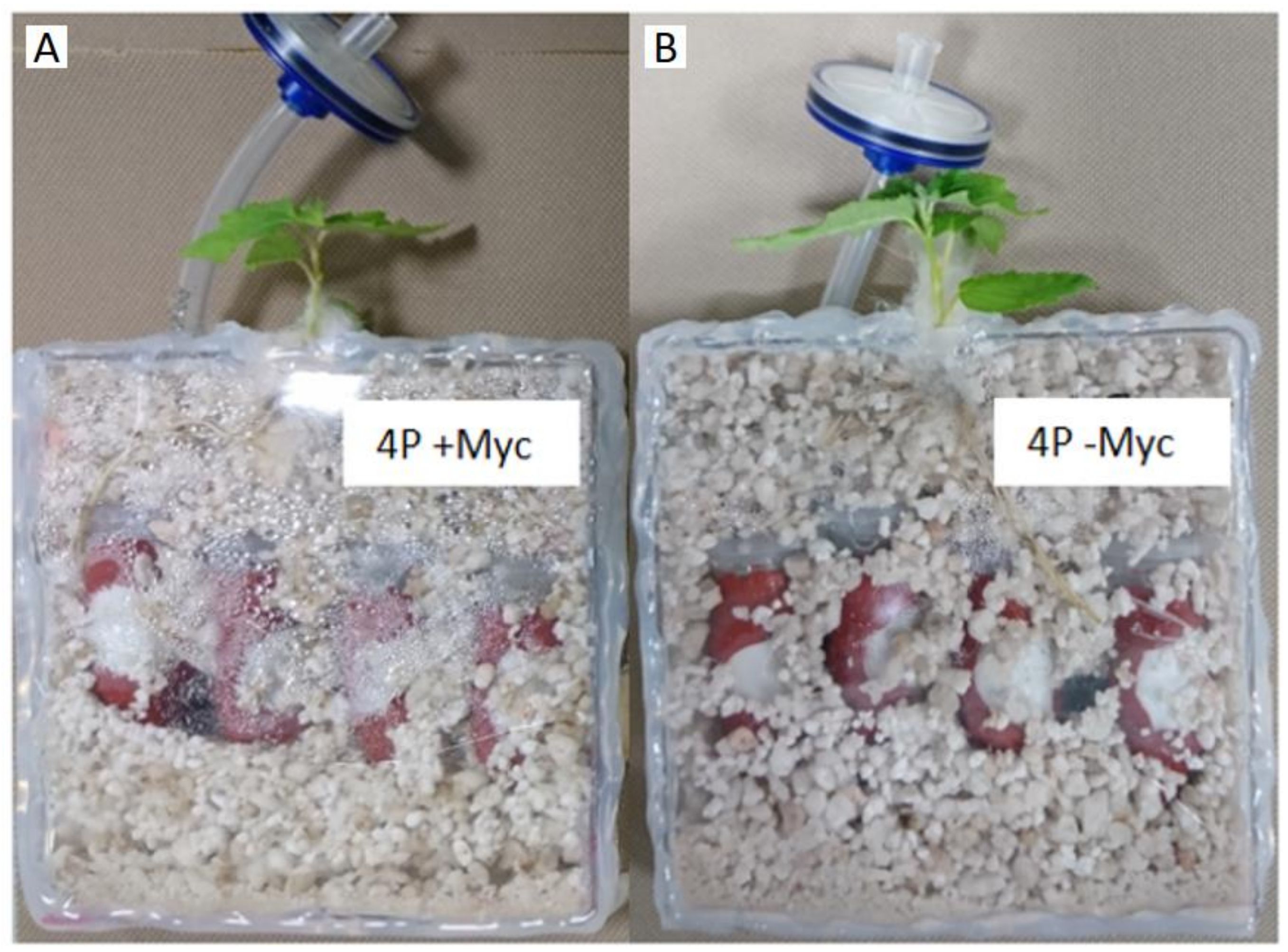

Supplement: Supplementary Figure 2 — Axenic rhizotrone culture system with four different chemical forms of P (4P) supplied in four separate hyphal compartments (HCs): the poplar plants Populus x canescence (Schleswig I) (A) mycorrhized (+Myc) by ectomycorrhizal fungus Paxillus involutus (MAJ) and (B) non-mycorrhizal (-Myc) control plants at 7 dpi. The HCs for the different P sources were made of 5 ml SafeSeal tubes and hydrophobic and nylon meshes. [file Image_2.jpg]

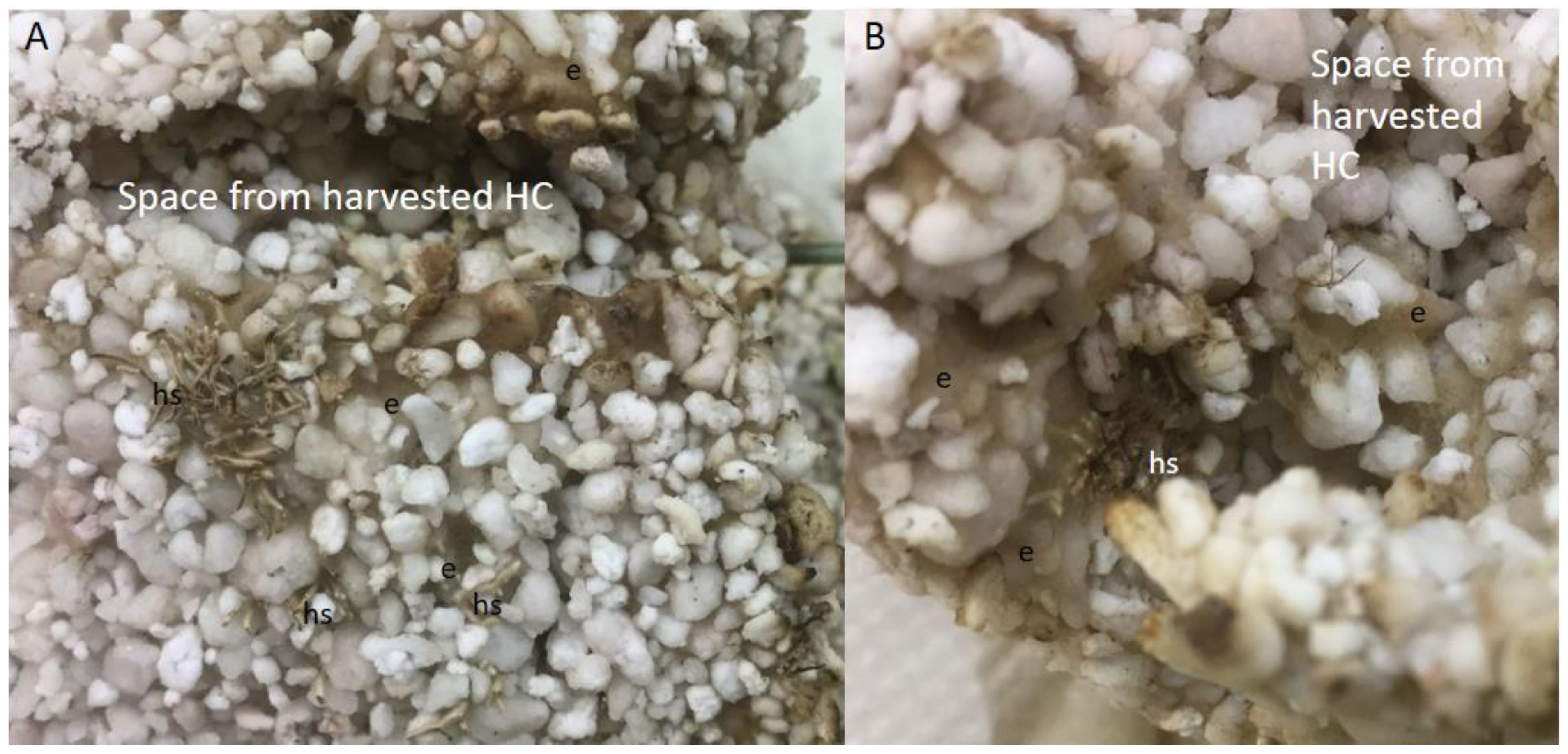

Supplement: Supplementary Figure 3 — Root tips of Populus x canescence (Schleswig I) colonised by the ectomycorrhizal fungus Paxillus involutus strain (MAJ) and its mycelium traversing the substrate, holding it as a block (at 126 dpi; viewed from (A) the side and (B) from above); hs: hyphal mantle around the root tips (mycorrhizal root tips); e: extraradical hyphae. [file Image_3.jpg]

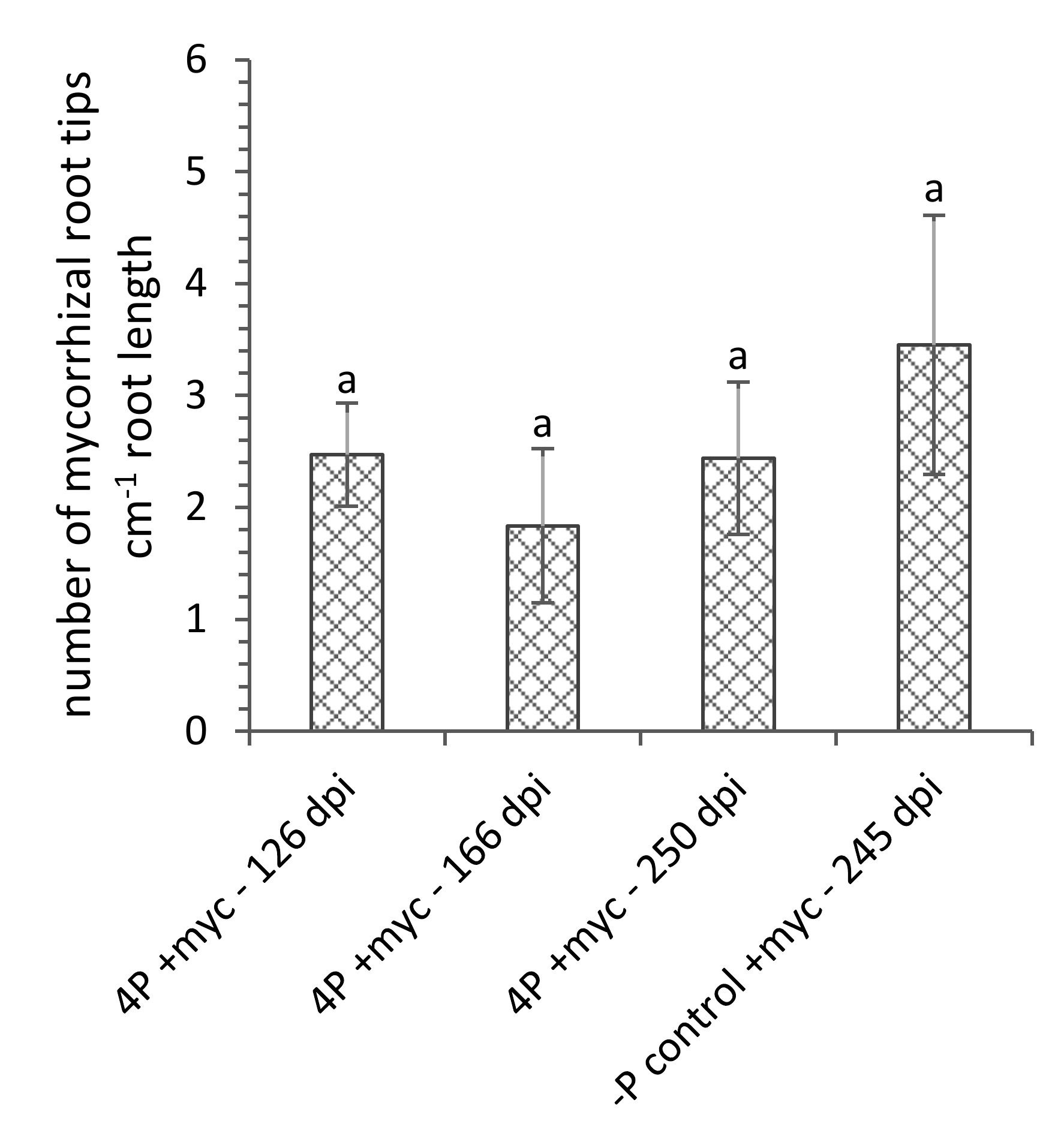

Supplement: Supplementary Figure 4 — Mycorrhization grade of plant roots expressed as mycorrhizal root tips per cm root length of Paxillus involutus ectomycorrhizal (+myc) roots of poplar plants Populus x canescens from different harvesting time points (126, 166, 250, and 243 dpi). The root length was determined using the grid-line intersection method (Giovannetti and Mosse, 1980; Brundrett et al. 1996). The error bars show the standard deviation (n=4). There was no significant (P < 0.05) difference in the mycorrhization grade of root tips between the different harvesting time points. [file Image_4.jpg]
